# Predictive Performance of NELA Versus P-POSSUM Mortality Scores: Are We Underestimating the Risk of Mortality Following Emergency Laparotomy?

**DOI:** 10.7759/cureus.32859

**Published:** 2022-12-23

**Authors:** Mohammed Barghash, Amir Iskandar, Sherif I Fawzy, Derek Effiom, Claire Huck, Shahin Hajibandeh, Shahab Hajibandeh, Moustafa Mansour

**Affiliations:** 1 Department of General Surgery, North Manchester General Hospital, Manchester University NHS Foundation Trust, Manchester, GBR; 2 Emergency Department, The Royal London Hospital, Barts Health NHS Trust, London, GBR; 3 Hepatobiliary and Pancreatic Surgery and Liver Transplant Unit, Queen Elizabeth Hospital, Birmingham, GBR; 4 Department of General Surgery, University Hospital of Wales, Cardiff, GBR

**Keywords:** score, mortality, laparotomy, p-possum, nela

## Abstract

Background

In this study, we aimed to compare the performance of the National Emergency Laparotomy Audit (NELA) and Portsmouth Physiological and Operative Severity Score for the enumeration of Mortality and Morbidity (P-POSSUM) mortality risk scores in predicting 30-day and 90-day mortality in patients undergoing emergency laparotomy.

Methodology

A retrospective cohort study was conducted to compare the predictive performance of preoperative NELA, postoperative NELA, and P-POSSUM predicted mortality scores in patients undergoing emergency laparotomy between 2014 and 2021. The outcomes of interest included the observed 30-day and 90-day mortality. The discrimination of the mortality tools was assessed and compared by determining the area under the curve (AUC) for each tool using the receiver operating characteristic curve analysis.

Results

A total of 681 patients were included. The observed risk of 30-day and 90-day mortality was 10.4% (71/681) and 14.2% (97/681), respectively. Regarding 30-day mortality, the AUC was 0.791 (0.727-0.855) for the preoperative NELA score, 0.784 (0.721-0.848) for the preoperative P-POSSUM score, and 0.761 (0.699-0.824) for the postoperative NELA score. Regarding 90-day mortality, the AUC was 0.765 (0.708-0.821) for the preoperative NELA score, 0.749 (0.692-0.807) for the preoperative P-POSSUM score, and 0.745 (0.691-0.800) for the postoperative NELA score. The observed/expected ratio for 30-day and 90-day mortality was 3.25 and 4.43 for preoperative NELA, 2.81 and 3.84 for preoperative P-POSSUM, and 2.17 and 2.96 for postoperative NELA, respectively. Pairwise comparisons showed no statistically significant difference in discrimination among the three models.

Conclusions

Preoperative NELA, postoperative NELA, and P-POSSUM scores underestimated the risk of 30-day and 90-day mortality in patients undergoing emergency laparotomy. No significant difference in predictive performance was found among the three models.

## Introduction

Emergency laparotomy is associated with a high risk of mortality, almost 10 times greater than that of major elective gastrointestinal surgery [1,2]. In addition to the operation-related factors, many patient-related factors also contribute to the outcomes of emergency laparotomy such as age, comorbidities, performance status, frailty, sarcopenia, and presence of peritoneal contamination [3-5]. Such factors need to be carefully addressed preoperatively to assess the likelihood of survival after emergency laparotomy. Consequently, to facilitate more objective decision-making, several scoring systems aiming at predicting surgical outcomes have been developed [6-9].

The Portsmouth Physiological and Operative Severity Score for the enumeration of Mortality and Morbidity (P-POSSUM) model and the National Emergency Laparotomy Audit (NELA) mortality risk score are two of the most popular risk prediction modalities [7,8]. The P-POSSUM was initially developed as the Physiological and Operative Severity Score for the enumeration of Mortality and Morbidity (POSSUM) by Copeland et al. [6] in 1991 and revised to P-POSSUM by Prytherch et al. in 1998 [7]. Although several studies have mentioned that P-POSSUM is an accurate predictor of mortality, there have been concerns about inaccuracy and potential overestimation of mortality in some subgroups of patients [10,11]. The NELA mortality risk score was developed using the large NELA database by the NELA team [8]. The model includes risk factors that are routinely collected in clinical practice and has demonstrated promising performance in calibration and discrimination [8].

Comparison of the predictive performance of the P-POSSUM model and NELA mortality risk score has been a subject of interest in recent studies [12,13]; however, the available comparative evidence is still limited. To add to the limited available evidence on the aforementioned comparison, we aimed to compare the performance of the P-POSSUM score and NELA mortality risk score in predicting 30-day and 90-day mortality in patients undergoing emergency laparotomy.

## Materials and methods

Participants, Interventions, Comparisons, Outcomes (PICO) research question

Is there any difference in the performance of NELA and P-POSSUM mortality scores in predicting 30-day and 90-day postoperative mortality in patients undergoing emergency laparotomy?

Study design and patient selection

We conducted a retrospective cohort study in compliance with the Strengthening the Reporting of Cohort Studies in Surgery (STROCSS) guidelines for observational studies [14]. The study followed a predefined protocol that was compliant with the institutions’ policies recommended by the local Clinical Governance Unit. Considering that the study had a retrospective design that used non-identifiable hospital data, there was no need for Research Ethics Committee approval and patient consent. The study was conducted in a General Surgery Department located in the North West of England. All patients aged over 18 who underwent emergency laparotomy between January 2014 and January 2021 were identified from a prospectively maintained electronic hospital database and included in the study. Patients who did not have all the relevant preoperative investigations required to calculate both NELA and P-POSSUM mortality scores were excluded from the study.

Comparisons

We aimed to synthesize outcomes for the following pairwise comparisons: (1) Preoperative NELA score versus preoperative P-POSSUM score. (2) Preoperative NELA score versus postoperative NELA score. (3) Preoperative P-POSSUM score versus postoperative NELA score.

Outcomes

The outcomes of this study included 30-day and 90-day postoperative mortality defined as death due to any cause occurring within 30 and 90 days following emergency laparotomy, respectively.

Data collection

A comprehensive electronic data collection proforma was developed to collect the following data: patients’ age, gender, American Society of Anesthesiology (ASA) score, clinical urgency, parameters required for calculation of NELA and P-POSSUM scores, 30-day mortality, and 90-day mortality.

Data synthesis and statistical analyses

The categorical variables were summarized using absolute and relative frequencies and compared using the chi-square test. The continuous variables were summarized using the median (minimum-maximum) and compared using the Mann-Whitney U test. For each predictive model, we calculated the observed deaths to expected deaths ratio (O/E ratio) by dividing the actual mortality rate (observed) by the calculated mortality score (expected). The O/E ratio would help assess whether the risk-prediction tool overestimates or underestimates the risk of postoperative mortality. The discrimination of the mortality tools was assessed and compared by determining the area under the curve (AUC) for each tool using receiver operating characteristic (ROC) curve analysis, as described by DeLong et al. [15]. All statistical tests were two-tailed and statistical significance was assumed at p-values of <0.05. The statistical analyses were performed using SPSS Statistics version 25 (IBM Corp., Armonk, NY, USA).

## Results

Baseline patient characteristics

A total of 743 patients underwent emergency laparotomy between 2014 and 2021 in our center, of whom 62 patients were excluded because they did not have all the relevant investigations required to calculate NELA or P-POSSUM mortality scores. As such, a total of 681 patients were included in the study. The median age of the included patients was 64 (18-91), and 45.4% (309 out of 681) were male. The ASA score was classified as 1 in 9.5% (65 out of 681), 2 in 35.2% (240 out of 681), 3 in 36.4% (248 out of 681), 4 in 16.6% (113 out of 681), and 5 in 2.2% (15 out of 681) (Table 1).

**Table 1:** Baseline characteristics of the included population in relation to 30-day and 90-day mortality. SD = standard deviation; ASA = American Society of Anesthesiologists; NCEPOD = National Confidential Enquiry into Patient Outcome and Death; NELA = National Emergency Laparotomy Audit; P-POSSUM = Portsmouth Physiological and Operative Severity Score for the enumeration of Mortality and Morbidity

	Total	Mortality in 30 days		Mortality in 90 days	
	681	No 89.6% (n = 610)	Yes 10.4% (n = 71)	No 85.8% (n = 584)	Yes 14.2% (n = 97)
Age on arrival					
Mean ± SD	61.15 ± 17.503	60.04 ± 17.682	70.70 ± 12.349	59.80 ± 17.770	69.29 ± 13.201
Median (Minimum–Maximum)	64 (18–91)	63 (18–91)	73(37–91)	63 (18–91)	71 (19–91)
Mann-Whitney U test (P)		29.214 (p < 0.001)		36.978 (p < 0.001)	
Sex					
Male	309 (45.4%)	275 (89.0%)	34 (11.0%)	264 (85.4%)	45 (14.6%)
Female	372 (54.6%)	335 (90.1%)	37 (9.9%)	320 (86.0%)	52 (14.0%)
Chi-square test (P)		0.202 (p = 0.653)		0.047(p = 0.828)	
ASA score					
1	65 (9.5%)	65 (100.0%)	0 (0.0%)	65 (100.0%)	0 (0.0%)
2	240 (35.2%)	233 (97.1%)	7 (2.9%)	227 (94.6%)	13 (5.4%)
3	248 (36.4%)	227 (91.5%)	21 (8.5%)	213 (85.9%)	35 (14.1%)
4	113 (16.6%)	79 (69.9%)	34 (30.1%)	74 (65.5%)	39 (34.5%)
5	15 (2.2%)	6 (40.0%)	9 (60.0%)	5 (33.3%)	10 (66.7%)
Chi-square test (P)		109.330 (p < 0.001)		97.865 (p < 0.001)	
NCEPOD urgency					
1	58 (8.5%)	41 (70.7%)	17 (29.3%)	39 (67.2%)	19 (32.8%)
2	500 (73.4%)	457 (91.4%)	43 (8.6%)	441 (88.2%)	59 (11.8%)
3	123 (18.1%)	112 (91.1%)	11 (8.9%)	104 (84.6%)	19 (15.4%)
Chi-square test (P)		24.223(p < 0.001)		18.868 (p < 0.001)	
Mortality scores					
Preoperative NELA					
Median (Minimum–Maximum)	3.2 (0.01–84.9)	2.7 (0.01–75.3)	17.2 (0.6–84.9)	2.6 (0.01–75.3)	15.4 (0.6–84.9)
Mann-Whitney U test (P)		34.274 (p < 0.001)		43.311 (p < 0.001)	
Preoperative P-Possum					
Median (Minimum–Maximum)	3.7 (0.01–96.7)	3.2 (0.01–96.7)	17.9 (0.59–87)	3.1 (0.01–96.7)	15.1 (0.59–87)
Mann-Whitney U test (P)		33.964 (p < 0.001)		42.441 (p < 0.001)	
Postoperative NELA					
Median (Minimum–Maximum)	4.8 (0.01–98.7)	4.4 (0.01–98.7)	23.7 (0.12–98)	4.1 (0.01–98.7)	17.1 (0.12–98)
Mann-Whitney test U (P)		32.977 (p < 0.001)		42.230 (p < 0.001)	

Postoperative mortality

Observed Mortality

The observed risk of 30-day mortality and 90-day mortality was calculated as 10.4% (71 out of 681) and 14.2% (97 out of 681), respectively.

Expected Mortality

The expected mortality risk was estimated as 3.2% (0.01-84.9) based on the preoperative NELA score, 3.7% (0.01-96.7) based on the preoperative P-POSSUM score, and 4.8% (0.01-98.7) based on the postoperative NELA score.

Observed/Expected Ratio

The O/E ratio for 30-day mortality was calculated as 3.25 for preoperative NELA, 2.81 for preoperative P-POSSUM, and 2.17 for postoperative NELA. The O/E ratio for 90-day mortality was calculated as 4.43 for preoperative NELA, 3.84 for preoperative P-POSSUM, and 2.96 for postoperative NELA. The above findings highlighted that all models underestimated the risks of 30-day mortality and 90-day mortality (Table 2).

**Table 2:** Observed deaths/Expected deaths ratio and AUC of the three tools for 30-day and 90-day mortality. O/E ratio = observed deaths/expected deaths; AUC = area under the curve; Std. Error = standard error; CI = confidence interval; Pre-op = preoperative; Post-op = postoperative; NELA = National Emergency Laparotomy Audit; P-POSSUM = Portsmouth Physiological and Operative Severity Score for the enumeration of Mortality and Morbidity

	Observed deaths	Expected deaths	O/E ratio	AUC	Std. error	P-value	95% CI	
							Lower bound	Upper bound
30-day mortality								
Pre-op NELA	10.4	3.2	3.25	0.791	0.033	<0.001*	0.727	0.855
Pre-op P-Possum	10.4	3.7	2.81	0.784	0.032	<0.001*	0.721	0.848
Post-op NELA	10.4	4.8	2.17	0.761	0.032	<0.001*	0.699	0.824
90-day mortality								
Pre-op NELA	14.2	3.2	4.43	0.765	0.029	<0.001*	0.708	0.821
Pre-op P-Possum	14.2	3.7	3.84	0.749	0.029	<0.001*	0.692	0.807
Post-op NELA	14.2	4.8	2.96	0.745	0.028	<0.001*	0.691	0.800

Pairwise comparisons

30-Day Mortality

**Figure 1:**
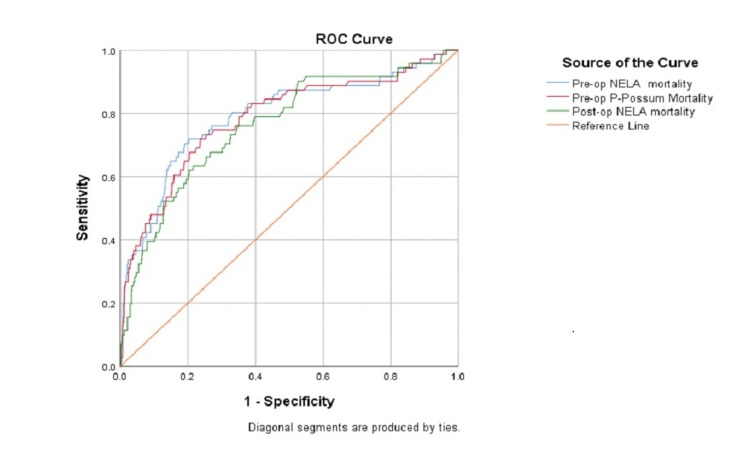
ROC curve of the three tools against observed 30-day mortality. Pre-op = preoperative; Post-op = postoperative; NELA = National Emergency Laparotomy Audit; P-POSSUM = Portsmouth Physiological and Operative Severity Score for the enumeration of Mortality and Morbidity; ROC = receiver operating characteristic

Preoperative NELA score versus preoperative P-POSSUM score: ROC curve analysis showed that the AUC for preoperative NELA score was 0.791 (95% confidence interval (CI) = 0.727-0.855, p < 0.001), and the AUC for the preoperative P-POSSUM score was 0.784 (95% CI = 0.721-0.848, p < 0.001). There was no significant difference in discrimination between the two scores (p = 0.594) (Figure 1 and Table 3).

**Table 3:** Pairwise comparison of the AUC for the three mortality scores against observed 30-day and 90-day mortality. AUC = area under the curve; Std. Error = standard error; CI = confidence interval; Pre-op = preoperative; Post-op = postoperative; NELA = National Emergency Laparotomy Audit; P-POSSUM = Portsmouth Physiological and Operative Severity Score for the enumeration of Mortality and Morbidity

	Difference between areas	Std. error	95% CI		Z statistic	P-value
			Lower bound	Upper bound		
30-day mortality						
Pre-op NELA vs. Pre-op P-Possum	0.00716	0.0134	-0.0191	0.0335	0.533	0.5938
Pre-op NELA vs. Post-op NELA	0.0299	0.0229	-0.0149	0.0748	1.309	0.1906
Pre-op P-Possum vs. Post-op NELA	0.0228	0.0215	-0.0194	0.0650	1.058	0.2899
90-day mortality						
Pre-op NELA vs. Pre-op P-Possum	0.0154	0.0112	-0.00659	0.0373	1.372	0.1702
Pre-op NELA vs. Post-op NELA	0.0191	0.0198	-0.0197	0.0579	0.963	0.3353
Pre-op P-Possum vs. Post-op NELA	0.00373	0.0199	-0.0352	0.0427	0.188	0.8509

Preoperative NELA score versus postoperative NELA score: ROC curve analysis showed that the AUC for the preoperative NELA score was 0.791 (95% CI = 0.727-0.855, p < 0.001), and the AUC for the postoperative NELA score was 0.761 (95% CI = 0.699-0.824, p < 0.001). There was no significant difference in discrimination between the two scores (p = 0.191) (Figure 1 and Table 3).

Preoperative P-POSSUM score versus postoperative NELA score: ROC curve analysis showed that the AUC for the preoperative P-POSSUM score was 0.784 (95% CI = 0.721-0.848, p < 0.001), and the AUC for the postoperative NELA score was 0.761 (95% CI = 0.699-0.824, p < 0.001). There was no significant difference in discrimination between the two scores (p = 0.289) (Figure 1 and Table 3).

90-Day Mortality

**Figure 2:**
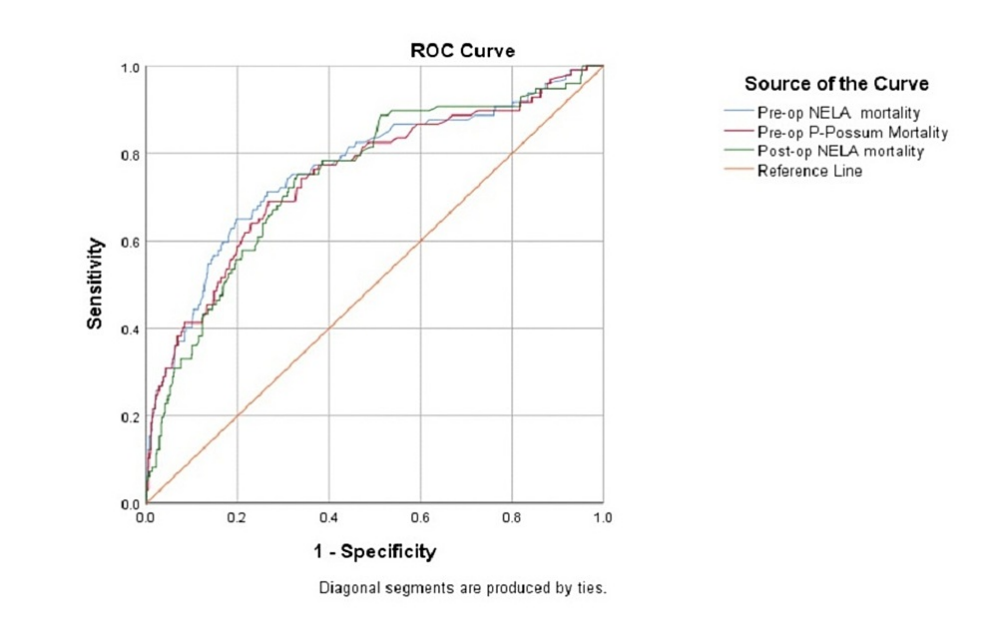
ROC curve of the three tools against the observed 90-day mortality. Pre-op = preoperative; Post-op = postoperative; NELA = National Emergency Laparotomy Audit; P-POSSUM = Portsmouth Physiological and Operative Severity Score for the enumeration of Mortality and Morbidity; ROC = receiver operating characteristic

Preoperative NELA score versus preoperative P-POSSUM score: ROC curve analysis showed that the AUC for the preoperative NELA score was 0.765 (95% CI = 0.708-0.821, p < 0.001), and the AUC for the preoperative P-POSSUM score was 0.749 (95% CI = 0.692-0.807, p < 0.001). There was no significant difference in discrimination between the two scores (p = 0.170) (Figure 2 and Table 3).

Preoperative NELA score versus postoperative NELA score: ROC curve analysis showed that the AUC for the preoperative NELA score was 0.765 (95% CI = 0.708- 0.821, p < 0.001), and the AUC for the postoperative NELA score was 0.745 (95% CI = 0.691-0.800, p < 0.001). There was no significant difference in discrimination between the two scores (p = 0.335) (Figure 2 and Table 3).

Preoperative P-POSSUM score versus postoperative NELA score: ROC curve analysis showed that the AUC for the preoperative P-POSSUM score was 0.749 (95% CI = 0.692-0.807, p < 0.001), and the AUC for the postoperative NELA score was 0.745 (95% CI = 0.691-0.800, p < 0.001). There was no significant difference in discrimination between the two scores (p = 0.851) (Figure 2 and Table 3).

## Discussion

We conducted a retrospective cohort study to compare the performance of the P-POSSUM model and NELA mortality risk score in predicting 30-day and 90-day mortality in patients undergoing emergency laparotomy. A total of 681 patients were included, and pairwise comparisons showed no significant difference in the predictive performance of preoperative NELA, preoperative P-POSSUM, and postoperative NELA scores in 30-day and 90-day mortality. All models underestimated the risks of 30-day mortality and 90-day mortality.

The performance of the NELA score and the POSSUM model have been compared in a few studies [12,13]. Thahir et al. [12] compared the performance of both models in 650 patients undergoing emergency laparotomy and concluded that the NELA model had better discriminative power than the P-POSSUM model in predicting 30-day mortality (AUC = 0.82 vs. 0.77). In the study by Thahir et al., the NELA mortality score underestimated the risk of mortality while the P-POSSUM overestimated the risk [12]. In another study, Lai et al. [13] compared the predictive performance of both scores in 830 patients and found no difference in the discriminative power of the NELA score and P-POSSUM model (AUC = 0.86 vs. 0.84). In the study by Lai et al., both models underestimated the risks of 30-day mortality [13]. The previous studies did not evaluate the predictive performance of the models for 90-day mortality. This might be because the NELA mortality score was initially developed to provide an estimate of the risk of death within 30 days of emergency abdominal surgery.

Taking into account the comparative evidence provided by the current study and previous studies, there is no robust or definite difference in predictive performance between the NELA model and the P-POSSUM model. Lai et al. suggested that the NELA score should replace the P-POSSUM model because the overestimation of mortality by the NELA score was less than the overestimation of mortality by the P-POSSUM model [13]. However, our results and the results by Thahir et al. do not support this rationale as in both studies NELA model underestimated the risk of mortality [12].

The necessity to improve the predictive performance of the NELA mortality score is absolute as it still underestimates both the 30-day and 90-day mortality following laparotomy. Hajibandeh et al. [9] compared the predictive performance of the Hajibandeh Index (HI), which is derived from combined levels of C-reactive protein, lactate, neutrophils, lymphocytes, and albumin, with the NELA mortality score in predicting postoperative 30-day and 90-day mortality and concluded that HI was better than the NELA score in predicting postoperative mortality in patients aged over 80 undergoing emergency laparotomy while its performance was comparable with the NELA score in other subgroups [9]. The authors argued that the comparable performance of the HI and NELA model suggests that the NELA model may not efficiently take into account the parameters that it asks for [9]. These findings may suggest that the current predictive tools do not take into account the modern predictors of mortality efficiently and may indicate that future studies should focus on the development and validation of scoring systems that take into account all of the modern predictors of mortality in patients undergoing emergency laparotomy such as HI [9], age over 80 [3,4], sarcopenia [5], ASA status above 3 [3,4], need for bowel resection [4], and presence of intraperitoneal contamination [3].

Several potential limitations can be found in this study. The current study had a retrospective design and its inherited limitations should be considered when interpreting its findings. The study was a single-center study; hence, it might not be the best representative of the entire population. We had to exclude 62 patients because they did not have all the relevant investigations required to calculate the NELA or P-POSSUM mortality scores; this should be taken into account when interpreting our findings. On the other hand, the use of an objective approach in protocoling, conduction, and reporting of this study; a relatively large sample size; consideration of 90-day mortality in addition to 30-day mortality; and evaluation of the performance of postoperative NELA mortality score alongside the performance of preoperative NELA and P-POSSUM scores were the strengths of this study.

## Conclusions

Based on pairwise comparisons, we demonstrated no significant difference in the predictive performance of preoperative NELA, preoperative P-POSSUM, and postoperative NELA scores in 30-day and 90-day mortality. All models underestimated the risks of 30-day mortality and 90-day mortality. Development and validation of scoring systems that take into account all of the modern predictors of mortality in patients undergoing emergency laparotomy should be the focus of future studies.
